# Physical and psychological states interfere with health-related quality of life of institutionalized elderly: a cross-sectional study

**DOI:** 10.1186/s12877-020-01791-6

**Published:** 2020-10-06

**Authors:** Ilky Pollansky Silva e Farias, Luiza de Almeida Souto Montenegro, Rayssa Lucena Wanderley, Jannerson Cesar Xavier de Pontes, Antonio Carlos Pereira, Leopoldina de Fátima Dantas de Almeida, Yuri Wanderley Cavalcanti

**Affiliations:** 1 Graduate Program in Dentistry, Federal University of Paraíba (UFPB), João Pessoa, PB Brazil; 2 Department of Social Dentistry, Piracicaba Dental School, University of Campinas (FOP-UNICAMP), Piracicaba, SP Brazil; 3 Department of Clinical and Social Dentistry, Federal University of Paraíba (UFPB), Campus I, Cidade Universitária, João Pessoa, PB 58051-900 Brazil

**Keywords:** Quality of life, Institutionalization, Aged, Frail elderly

## Abstract

**Background:**

Nursing home elders experience many problems that may influence their quality of life, in example of cognitive, mental, nutritional and physical disabilities. Concerning about elders’ wellbeing may help them living with dignity. This study aimed to investigate factors associated with Health-Related Quality of Life (HRQoL) of institutionalized elders in a capital city of Brazilian Northeast.

**Methods:**

A cross-sectional study was conducted with 125 institutionalized elders living in the metropolitan region of João Pessoa (Brazil). The following variables were tested regarding their association with the elders’ HRQoL: Socio-demographic characteristics; Performance of daily-living activities, Frailty status, Cognitive status, Nutritional status, Self-perception of oral health and Depression status. Hierarchical multiple Poisson loglinear and binary logistic regressions analyses were performed in order to assess the impact of each independent variable on HRQoL, considering a significance level of 5%.

**Results:**

The median of HRQoL of institutionalized elders was 64. Multivariate regression models showed that retirement, frailty and depression were statistically associated with poor HRQoL (*p* < 0.05). Not-frail elderly and less depressed were more likely to present higher HRQoL scores.

**Conclusions:**

Lower HRQoL of institutionalized elderly is associated with decline of physical and psychological states. Institutions should be advised to plan and implement actions that would improve the HRQoL of institutionalized elderly.

## Background

A significant demographic transition has been experienced worldwide due to declining birth rates and increasing life expectancy, leading to an increase in the elderly population [1]. This phenomenon is particularly progressing fast in Brazil, and this may undertake Brazilian elderly population to rank sixth in 2020 [2]. Based on that, researchers have discussed and investigated the mechanisms associated with aging, especially the socio-cultural, psychological, and economic implications that involve this process. Although the population aging is a positive phenomenon, it also imposes many important public health challenges [3, 4].

The World Health Organization (WHO) defines quality of life as the individual’s perception of their position in life according to their culture and value system, which is affected in a complex way by the person’s physical and psychological health, social relationships and personal beliefs [5]. In health sciences, the term ‘quality of life’ usually refers to how the individual’s wellbeing may be affected by a disease or disability [6]. The Health-Related Quality of Life (HRQoL) is not a single entity, but a complex state comprised of several domains, including physical, emotional, spiritual, cognitive and social wellbeing [7]. In addition, socioeconomic and socio-demographic aspects of the social environment can strongly influence the HRQoL [8].

Increased life expectancy is associated with an increased age-related vulnerability and disability [2]. Although it is expected a greater susceptibility of the elder to physical and cognitive problems, strategies may be implemented to reduce the impact of those health problems on their functional status, autonomy and independence [9]. Increasing prevalence of chronic diseases, such as depressive symptoms, mental health problems, polypharmacy and the presence of geriatric syndromes (eg. dependence on daily-living activities, recurrent falls and urinary incontinence) are strongly associated with the increased rate of institutionalization [10].

Some factors may be associated to quality of life worsening of institutionalized elders, including: loss of freedom and privacy, absence of family and friends, and feeling of abandonment. Reduction or even loss of functional capacity and decrease of cognitive abilities, such as memory and learning, may also worsen these individual’s living conditions. Another relevant point is that daily activities are often carried out in the same environment, being the routine equal for all, a situation that contributes to the development of symptoms of depression and anxiety [11]. In this context, the worsening of health conditions is often related to elderly’s institutionalization [12, 13]. As many of institutionalized elders face cognitive decline, few is known about their health-related quality of life and its associated factors.

Based in this context and considering that institutionalization process has been related to a higher prevalence of comorbidities, including a significant functional and cognitive decline [14], identifying factors that can impact the quality of life may be a useful tool for the implementation of health promotion and prevention strategies for institutionalized elderly. A recent meta-analysis demonstrated that institutionalized elders have worse quality of life compared to home dwelling ones [15]. Considering the high concern with the quality of life of this population and the challenges in promoting elderly the opportunity to live with dignity, the present study aimed to determine factors related to HRQoL of institutionalized elderly in a capital city of Brazilian Northeast. Study’s null hypothesis is that socio-demographic and health-related variables are not associated with HRQoL of institutionalized elderly.

## Methods

### Ethical aspects

The Ethics Research Committee from Federal University of Paraiba approved this study (CAAE: 66122917.6.0000.5188), in accordance with the ethical standards of the Brazilian Ministry of Health, as well as with the 1964 Helsinki declaration and its later amendments. All participants gave a free written informed consent.

### Research scenario

This study was carried out in a capital city of Brazilian Northeast (João Pessoa, Paraíba, Brazil - Latitude: 07° 06′ 54“ S, Longitude: 34° 51’ 47” W). This city has 800,323 inhabitants, a human development index at 0.763 and a per capita gross domestic product of R$23,169 (roughly US$ 6400). Within the metropolitan region of João Pessoa, there are seven long-term care institutions, which assist an average of 50 elders per institution. Institutions are philanthropic and most of costs are covered by elders’ income (average US$ 200 per month).

### Subjects and study design

Institutionalized elderly population in the metropolitan region of João Pessoa consisted of 398 individuals. In a pilot study, we detected that the response rate was around 40%, since many of the institutionalized individuals had seriously cognitive impairment. A design effect of 1.4 was calculated and the sample size of 191 was set as representative of non-to-middle cognitive impaired elderly.

A cross-sectional investigation was then conducted with 193 institutionalized older adults living in seven long-term care institutions located in a capital city of Brazilian Northeast. Inclusion criteria required elderly to assimilate the methodological tools and agree to participate in the survey. Initial screening of participants was achieved after subjective evaluation and assessment of cognitive status with regards to space-time orientation. The Mini-Mental State Examination (MMSE) was used for including or not individuals within the study. A minimum of 18 points for illiterate individuals and a minimum of 21 points for literate ones were considered as inclusion criteria [16, 17]. In addition, subjects with chronic degenerative diseases (i.e. Azheimer disease and Parkinson disease) were not included within the study. Subjects answered the questionnaires after agreeing participate in the research and sign an informed consent. Based on the cut-off point of (MMSE), the final sample size considered for analysis was equivalent to 125.

Seven previously trained researchers took part in this survey. Training of researchers involved a theoretical exposition of all validated instruments, as well as a clinical experience to set the collection procedure. Concordance was set within the group of examiners as above 0.9.

### Questionnaire and variables

The following independents variables were included in the present study: socio-demographic characteristics (sex, age, educational level, retirement and family visits) and data associated with general health (Performance of daily-living activities, Frailty status, Cognitive status, Nutritional status, Self-perception of oral health and Depression status). HRQoL was considered the dependent variable in this study. All data were collected using validated questionnaires, which were used to interview the subjects. None of the questionnaires used require a license to administer them.

### Performance of daily-living activities (Katz scale)

The performance of daily-living activities was assessed using a six items questionnaire that measured the individual’s performance in self-care activities, including the following domains: 1) feeding; 2) sphincter control; 3) transference; 4) personal hygiene and use of the toilet; 5) dressing ability; 6) taking a shower [18]. Each dependence score was considered one point. In this survey, participants who were dependent of two or more functions were considered dependent.

### Frailty status

Frailty status was evaluated using a self-reported questionnaire validated by Nunes et al. [19] and adapted from the original proposed by Fried et al. [20]. The questionnaire includes five criteria: non-intentional weight loss, poor energy and endurance, muscle force reduction, sedentary behaviour, and slowness. In this survey, participants were categorized as frail when they have three or more positive scores for frailty. Muscle force was evaluated using a handgrip dynamometer; however, the handgrip force was not included in the statistical model.

### Cognitive status

The Mini-Mental State Examination (MMSE) was used to assess the cognitive impairment and used as a screening for dementia [21]. The MMSE is composed of typical questions grouped into seven categories, each of which aims to evaluate specific cognitive functions: orientation to space-time, attention and calculation, word registration, language, word recall, and visual construction [22]. The MMSE score ranges from 0 to 30; cut-off points of 18 and 21 points within MMSE were used as inclusion criteria for illiterate and literate individuals, respectively [16, 17]. In this survey, total MMSE score (ranging from 18 to 30) was used for analysis.

### Nutritional status

The Mini Nutritional Assessment Short Form (MNA-SF) is a well-validated technique that evaluates the risk of malnutrition among elders, by means of a self-reported instrument. The MNA-SF is based on an assessment of general health and on a self-perception of health and nutrition. It also included the calf circumference assessment. MNA-SF scores ranges from 0 to 14 and higher score indicated a more satisfactory state of nutrition [23]. In this survey, total MNA-SF score was used for analysis.

### Self-perceived oral health

The elders’ self-perception on oral health was assessed using the Geriatric Oral Health Assessment Index (GOHAI). For this, a 12 items questionnaire analyzed the physical function, psychosocial function and pain or discomfort [24]. Voluntaries answered questions as never (score 3), sometimes (score 2) and always (score 1). GOHAI scores ranges from 12 to 36 and higher score indicate better self-perception on oral health. In this survey, total GOHAI score was used for analysis.

### Depression status

The Geriatric Depression Scale (GDS) instrument was used to assess the depression status among institutionalized elders. GDS instrument consists 15 items questionnaire that is validated to older adult populations [25, 26]. Each item can have 2 answers (yes or no). The highest possible score is 15, which indicates the most severe depressive state [27]. In this survey, total GDS score was used for analysis.

### Health-related quality of life (HRQoL)

HRQoL was measured using the SF-12 instrument, which consists a 12 items questionnaire that assessed the following concepts: general health, vitality, physical functioning, physical impairment, pain, emotional health problems, mental health problems, and social activity [28]. HRQoL scores ranges from 0 to 100 and higher scores indicate better HRQoL. Data from HRQoL was used in continuous form (total score), and categorized according to the median. In this survey, participants were included in two categories, according to the median value found in this study: poor HRQoL (< 64 points) or good HRQoL (≥ 64 points).

#### Theoretical-conceptual model

**Fig. 1:**
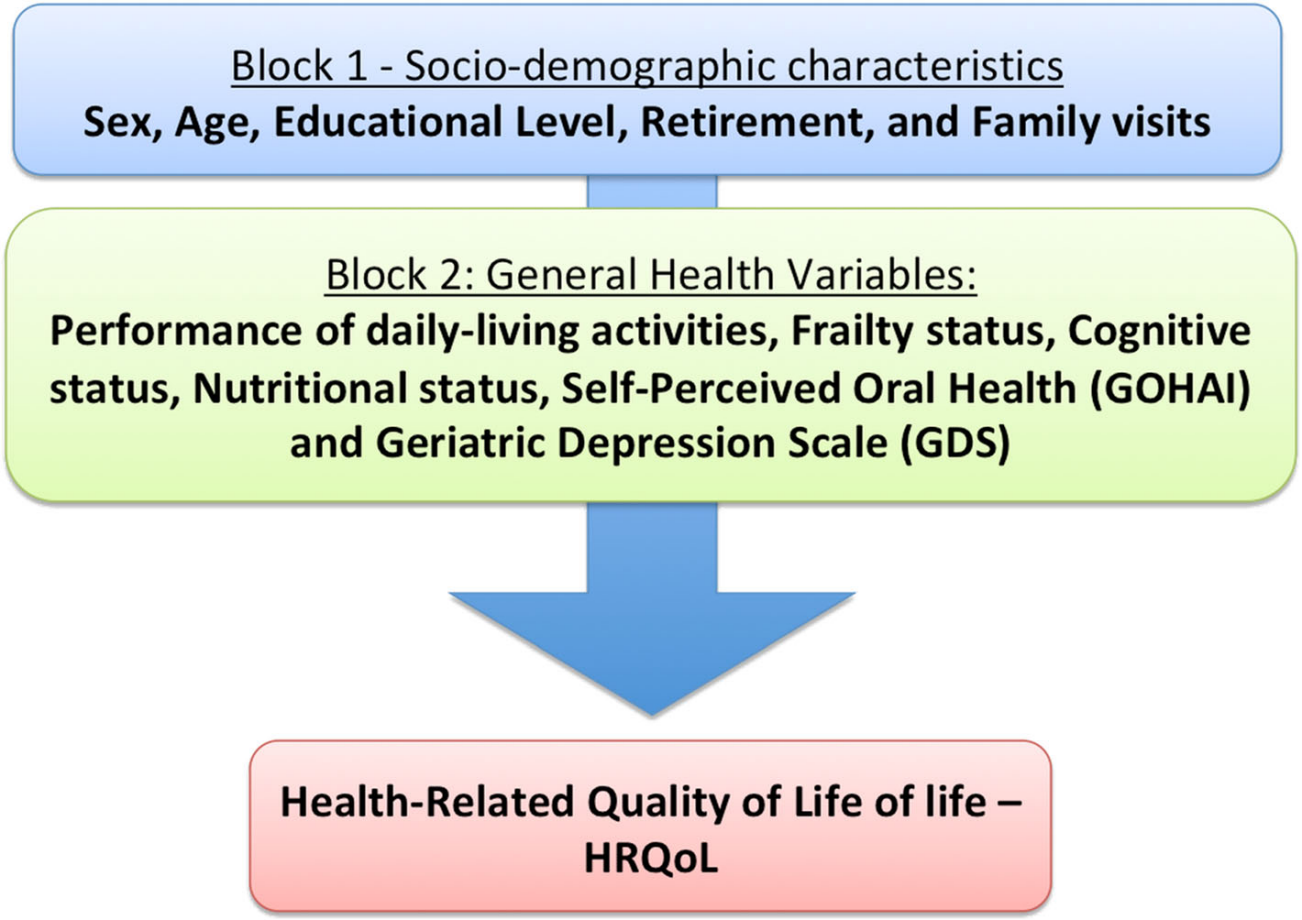
Theoretical-conceptual model of factors that would determine the Health-Related Quality of Life (HRQoL – dependent variable) of institutionalized elderly

A theoretical-conceptual model was designed to determine factors related to HRQoL of institutionalized elderly involved in this survey (Fig. 1). Block 1 included independent variables related to social status (sex, age, retirement and family visits). Block 2 included independent variables related to general health (Performance of daily-living activities, Frailty status, Cognitive status and Nutritional status, Self-perception of oral health and Geriatric Depression Scale).

#### Statistical analysis

Descriptive analyses were conducted to check absolute and relative distributions, as well as to calculate means, medians and standard deviations. Data were analyzed using IBM Statistical Package for Social Sciences software (IBM SPSS, v. 20, Chicago, IL). The independent variables were evaluated with regards their association with HRQoL through the use of two statistical regression models (multiple Poisson loglinear and multiple binary logistic), as shown in Fig. 1. Bivariate associations with categorical variables were assessed using chi-square and exact Fisher tests (*p* < 0.05). For multiple Poisson loglinear regression model, the HRQoL scores was used under its continuous form. For multiple binary logistic model, HRQoL scores were dichotomized according to the median. No missing data was detected in this study. Initially, all variables were included within the model, using the hierarchical approach presented in Fig. 1. Variables within each block were analyzed with regards their significance. Variables with *p*-value above 0.20 were progressively removed using the Backward-Wald method. Odds ratios (ORs) were reported with 95% confidence intervals (95% CI). A significance level of 5% was the criterion for a statistically significant effect. Models adjustments were assessed through Omnibus test, considering *p* < 0.05.

## Results

Out of 193 individuals initially screened, 68 presented MMSE scores bellow 18 and were excluded from the study. Final sample consisted of 125 institutionalized elders, from which 66.4% were female, 59.2% had at least primary school level, 86.4% were retired, and 72.8% received visits from their relatives (Table 1). In bivariate analysis, better HRQoL scores (≥ 64 points) was statistically associated with retirement, performance of daily-living activities frailty status (*p* < 0.05) (Table 1). The median age of elderly individuals was 79 (Table 2). Descriptive statistics of scores obtained for nutritional status (MNA-SF), cognitive status (MMSE), self-perceived oral health (GOHAI), depression status (GDS) and health-related quality of life (HRQoL) are presented in Table 2. Maximum HRQoL was 90 and 75% of individuals scored up to 78 points in HRQoL questionnaire.

**Table 1:** Frequency distribution of institutionalized elderly’s HRQoL (*n* = 125), according to sex, educational level, retirement, family visits, performance of daily-living activities and frailty status

Independent Variables		HRQoL				***p***-value*
		Poor (< 62 points)		Good (≥ 62 points)		
		n	%	n	%	
**Sex**	Male	21	16.8	21	16.8	0.848
	Female	40	32.0	43	34.4	
**Educational level**	Primary, secondary or higher education	36	28.8	39	31.2	0.827
	Literate or illiterate	25	20.0	25	20.0	
**Retirement**	Yes	59	47.2	51	40.8	0.002
	No	2	1.6	15	12.0	
**Family visits**	Yes	44	35.2	48	38.4	0.717
	No	17	13.6	16	12.8	
**Performance of daily-living activities**	Independent (dependent of ≤1 function)	43	34.4	58	46.4	0.004
	Dependent (dependent of ≥2 functions)	18	14.4	6	4.8	
**Frailty status**	Not Frail (< 3 points)	19	15.2	50	40.0	< 0.001
	Frail (≥ 3 points)	42	33.6	14	11.2	

*chi-square or exact Fisher test

**Table 2:** Descriptive statistics regarding age, nutritional status (MNA-SF), cognitive status (MMSE), self-perceived oral health (GOHAI), depression status (GDS) and health-related quality of life (HRQoL) of institutionalized elderly (*n* = 125)

Variables	Mean	SD	Median	Min.	Max.	Q25	Q75
**Age**	78.82	8.16	79	60	99	74	85
**Nutritional status (MNA-SF)**	9.82	3.65	11	2	14	8	13
**Cognitive status (MMSE)**	23.92	3.80	24	18	30	21	27
**Self-perceived oral health (GOHAI)**	32.10	4.15	33	0	37	31	34
**Depression (GDS)**	4.61	3.51	4	0	14	2	7
**Health-related quality of life (HRQoL)**	63.42	16.09	64	32	90	50	78

*SD* Standard Deviation, *Min*. Minimum value, *Max*. Maximum value, *Q25* 1st quartile (25%), *Q75* 3rd quartile (75%)

Multivariate Poisson loglinear regression model showed that retirement, frailty and depression states are statistically associated (*p* < 0.05) with the HRQoL score (Table 3). According to adjusted model, retired individuals are less likely to present higher HRQoL scores (OR = 0.870), whilst not-frail elderly are more likely to present higher HRQoL scores (OR = 1.144). Individuals with higher GDS scores were less likely to present higher HRQoL scores (OR = 0.966). Similarly to multivariate Poisson loglinear regression, multiple binary logistic adjusted regression showed negative influence of retirement, frailty and depression states on the proportion of individuals with HRQoL scores above the median (64 points) (Table 4).

**Table 3:** Crude and adjusted multiple Poisson loglinear regression models to predict variables associated with the HRQoL score among institutionalized elderly. Statistically significant linear regression coefficients (B) result in an impact on HRQoL score

**CRUDE MODEL**	**B**	**S.E.**	***p*-value**	**OR**	**95% C.I.**	
					**Lower**	**Upper**
**Age (years)**	0.000	0.0021	0.927	1.000	0.996	1.004
**Sex (male)**	0.028	0.0362	0.443	1.028	0.958	1.104
**Education (higher)**	−0.031	0.0379	0.419	0.970	0.900	1.045
**Retirement (yes)**	−0.159	0.0662	0.016	0.853	0.749	0.971
**Family visits (yes)**	0.067	0.0433	0.122	1.069	0.982	1.164
**Independent elderly**	0.015	0.0490	0.760	1.015	0.922	1.117
**Not-frail elderly**	0.139	0.0491	0.005	1.149	1.044	1.265
**MNA-SF score**	0.005	0.0052	0.336	1.005	0.995	1.015
**GOHAI score**	0.003	0.0035	0.401	1.003	0.996	1.010
**GDS score**	−0.034	0.0073	< 0.001	0.967	0.953	0.981
**MMSE score**	0.003	0.0049	0.589	1.003	0.993	1.012
ADJUSTED MODEL*	**B**	**S.E.**	***p*-value**	**OR**	**95% C.I.**	
					**Lower**	**Upper**
**Retirement (yes)**	**−0.139**	**0.0630**	**0.027**	**0.870**	**0.769**	**0.984**
**Family visits (yes)**	0.064	0.0405	0.115	1.066	0.985	1.154
**Not-frail elderly**	**0.135**	**0.0471**	**0.004**	**1.144**	**1.043**	**1.255**
**MNA-SF score**	0.007	0.0049	0.154	1.007	0.997	1.017
**GDS score**	**−0.035**	**0.0070**	**< 0.001**	**0.966**	**0.953**	**0.979**

* Model adjustment after progressive removal of variables: Age (*p* = 0.927), Dependency (0.761), MMSE (*p* = 0.566), Education (*p* = 0.527), Sex (*p* = 0.483), and GOHAI (*p* = 0.359). Omnibus test significance: *p* < 0.001

**Table 4:** Crude and adjusted Multiple binary logistic regression models to predict variables associated with good HRQoL (≥64 points) of institutionalized elderly. Variables with statistically significant Odds Ratio (OR) impacted negatively the HRQoL score

**CRUDE MODEL**	**B**	**S.E.**	***p*-value**	**OR**	**95% C.I.**	
					**Lower**	**Upper**
**Age (years)**	0.036	0.0346	0.299	1.037	0.969	1.109
**Sex (male)**	0.133	0.5169	0.797	1.142	0.415	3.146
**Education (higher)**	−0.694	0.6028	0.250	0.500	0.153	1.629
**Retirement (yes)**	−2.786	0.9543	0.004	0.062	0.010	0.400
**Family visits (yes)**	1.151	0.6742	0.088	3.161	0.843	11.849
**Independent elderly**	0.230	0.7914	0.771	1.259	0.267	5.939
**Not-frail elderly**	1.524	0.5551	0.006	4.589	1.546	13.622
**MNA-SF score**	0.089	0.0755	0.240	1.093	0.942	1.267
**GOHAI score**	0.023	0.0415	0.571	1.024	0.944	1.110
**GDS score**	−0.350	0.1073	0.001	0.705	0.571	0.870
**MMSE score**	0.056	0.0703	0.429	1.057	0.921	1.213
ADJUSTED MODEL*	**B**	**S.E.**	***p*-value**	**OR**	**95% C.I.**	
					**Lower**	**Upper**
**Retirement (yes)**	**−2.248**	**0.9418**	**0.017**	**0.106**	**0.017**	**0.669**
**Family visits (yes)**	0.968	0.5901	0.101	2.633	0.828	8.371
**Not-frail elderly**	**1.376**	**0.5066**	**0.007**	**3.959**	**1.467**	**10.686**
**MNA-SF score**	0.091	0.0677	0.180	1.095	0.959	1.250
**GDS score**	**−0.349**	**0.1098**	**0.001**	**0.705**	**0.569**	**0.874**

* Model adjustment after progressive removal of variables: Sex (*p* = 0.797), Dependency (*p* = 0.788), GOHAI (*p* = 0.570), MMSE (*p* = 0.410), Age (*p* = 0.357) and Education (*p* = 0.311). Omnibus test significance: *p* < 0.001

## Discussion

The results from this study point out retirement, frailty and depression are factors significantly associated with lower HRQoL of institutionalized elderly. Based on that, institutions should be advised to promote activities that could improve elderly functions and reducing frailty and depression. Better HRQoL is necessary for institutionalized elderly live with dignity.

Some studies emphasized that institutionalization process result in a worse HRQoL [15]. Although institutionalized elderly have lower HRQoL, this condition is possibly associated with factors that led them to institutionalization, such as very advanced age, low scholarship, low autonomy and low social participation [15, 29, 30].

It is evident that retirement is not a condition derived from institutionalization. Very little is possible to do at institutions’ level with regards to the retirement’s impact on HRQoL. The literature has reported a decrease in perceived HRQoL after retirement, and this is frequently associated with a decline in functional, physical, mental and emotional states [31–34]. In addition, the type of occupation during the course of life and the absence of functional activities in the elderly life are also factors that strengthen the relationship between retirement and poor HRQoL [33, 34]. Based on that, seems reasonable to recommend institutions improving the level of activities directed to elderly individuals. Avoiding the decline of functional, mental and emotional states would consequently impact the decline of HRQoL [32, 34].

Brazil has an important cultural diversity and socioeconomic inequalities, resulting in heterogeneous institutions for elderly with regards to provision of services, physical structure, financial resources and the strata of public served [35]. In this context, it is evident that the HRQoL experienced by different elders may vary within the same institution and among different places. However, this study point out that long-term care facilities may improve the way-of-life of their residents through physical and mental health promotion activities, which may impact positively their general HRQoL [36].

A substantial impact of physical, cognitive and psychological disabilities on HRQoL of institutionalized elderly has been demonstrated [37]. According to this previous study, providing psychological, physical and occupational interventions could significantly improve HRQoL of nursing home residents. The results of our study corroborate with those previously reported, since it was also detected a relationship between physical (frailty status) and psychological (depression status) states on HRQoL of institutionalized elderly.

The reduction in the body mass and loss of muscle tone are characteristics involved in aging process and these factors imply in the reduction of movements, decrease of functional performance and induction of frailty [38, 39]. The lack of physical stimulation in institutions increases the probability of functional disorders in elderly, which affects their HRQoL [40]. A previous study has demonstrated that lower levels of physical activity were associated with institutionalization [41].

In addition to a pre-existent condition, long-term care institutions frequently limit elderly’s active lifestyle due to the lack of sufficient personnel and infrastructure [15, 42]. In our study, none of the institutions investigated presented a regular program of physical activities for the elderly and this fact may be related to the reduced number of employees. In general, institutions have small number of professionals that are required to perform many functions, including the practice of regular physical activities among elderly.

Although this study did not show an impact of nutritional status on the HRQoL of institutionalized elderly, previous studies have shown that malnutrition may contribute to increased mortality and greater susceptibility to infections, which may reduce the elderly’s HRQoL [43, 44]. Other studies emphasize malnutrition is related to functional disability and frailty, as result of muscle strength decrease and reduction of cardiorespiratory performance [45, 46].

The poor heath status of institutionalized elderly increases the incidence of elderly’s mortality, hospitalization and institutionalization [47], which obviously impact the HRQoL. Poor nutritional status frequently accelerates the onset of frailty and predisposes elderly people to chronic diseases [48]. Therefore, frail elderly must be subjected to nutritional supplementation and physical activities in order to improve functional performance, nutritional status and the overall HRQoL [49].

The overall prevalence of frailty in the community-dwelling population of western countries has been reported to range from 6 to 40% [50–53]. We detected that 44.8% of participants were considered frail, and this impacted significantly the HRQoL of institutionalized elderly. Although there is evidence of the large benefits of exercising in improving functional and mental domains of elderly’s quality of life, no recommendations have been made to date concerning the structure of exercise programs directed to frail institutionalized elderly [54, 55]. None physical exercise programs were observed within the institutions visited in our study. Nevertheless, valid interventions for community-dwelling older adults are not necessarily valid for nursing home populations, since institutionalized elderly have higher rates of disability, multiple morbidities, and geriatric syndromes [49].

Depression is the most prevalent functional mental disorder in elderly people. It is projected that depressive illness will be the second leading cause of disability worldwide in 2020 [56]. The degree of unhappiness and suffering in people with depression is not easily measured, although one possible way is to assess the impact of depression on their quality of life. Even minor levels of depression have been related to a significant quality of life decrease among elders [57]. The results of this survey showed that depression status was associated with lower HRQoL among institutionalized elders. Developing programs for psychological monitoring and depression prevention are therefore necessary. This would aid reducing the negative effects of depression on HRQoL. Although GDS is not a valid instrument for diagnosing depression, it has an excellent applicability in long-terms care institutions. GDS can contribute to monitoring the prevalence of symptoms related to depression [58].

The relationship between depression and poor quality of life perception among elders has been demonstrated previously [59]. The loss of independence and privacy within long-term care institutions can aggravate the depression status among institutionalized elders [41]. Psychological illness is usually associated with lower life enjoyment and demotivation, which implies in lower functional capacity and lower quality of life [59].

The results of this study did not find statistical associations between geriatric self-perceived oral health (GOHAI) and HRQoL. The oral health of institutionalized elderly was previously characterized by high frequency of tooth loss, lack of regular preventive care and lack of dental treatment [60, 61]. This illustrates that oral health is undervalued among institutionalized elders. Therefore, the self-perceived oral health does not seem to impact the HRQoL of institutionalized elders. Nevertheless, improvement of elders’ oral health would possibly impact the masticatory function, nutritional status and self-esteem.

It is important to consider this is a cross-sectional study and statistically significant associations may not always represent a cause-effect relationship. Although the sample size can be considered limited, this was set by a statistical calculation and it represents the whole number of institutionalized elderly that could answer the validated questionnaires with certain level of reliability. The results of this study can be set as representative of institutions from the capital cities of Brazil Northeast, as well as other countries with similar economical status or long term care institutions structure. Results of this study could aid institutions to promote physical and psychological interventions to prevent frailty and depression among institutionalized individuals.

## Conclusions

In our study, retired, frail and depressed institutionalized elders presented a higher chance to have worse HRQoL. These findings emphasize the need to plan and implement strategies to impact significantly the HRQoL of institutionalized elders. In addition, the inclusion of physical activities programs and recreational activities may contribute positively to the recovery of the physical and mental states of these individuals, allowing them to live with dignity and better quality of life.

## Data Availability

The datasets used for this study is available from the corresponding author on reasonable request.

## Abbreviations

GDS: Geriatric Depression Scale
GOHAI: Geriatric Oral Health Assessment Index
HRQoL: Health-Related Quality of Live
MMSE: Mini-Mental State Examination
MNA-SF: Mini Nutritional Assessment – Short Form
OR: Odds Ratio
SD: Standard Deviation
WHO: World Health Organization
95%CI: 95% Confidence Interval
